# The impact of ionizing irradiation on liver detoxifying enzymes. A re-investigation

**DOI:** 10.1038/s41420-019-0148-8

**Published:** 2019-02-08

**Authors:** Alessio Bocedi, Gianluca Ingrosso, Giada Cattani, Roberto Miceli, Elisabetta Ponti, Andrea Lancia, Sara Baldelli, Arianna Guidi, Maria Rosa Ciriolo, Maurizio Mattei, Giorgio Ricci

**Affiliations:** 1 Department of Chemical Sciences and Technologies, University of Rome “Tor Vergata”, Rome, Italy; 2 Department of Diagnostic Imaging, Molecular Imaging, Interventional Radiology and Radiotherapy, Tor Vergata General Hospital, Rome, Italy; 3 IRCCS San Raffaele “La Pisana”, Rome, Italy; 4 Department of Biology, University of Rome “Tor Vergata”, Rome, Italy; 5 Interdepartmental Center for Animal Technology, University of Rome “Tor Vergata”, Rome, Italy

## Abstract

By looking at many studies describing the impact of ionizing irradiations in living mice on a few key detoxifying enzymes like catalase, superoxide dismutase, glutathione peroxidase, glutathione reductase and glutathione transferase, we noted conflicting evidences: almost all papers finalized to demonstrate the protective effects of natural or synthetic drugs against the damage by irradiations, described also a relevant inactivation of these enzymes in the absence of these compounds. Conversely, no inactivation and even enhanced activity has been noted under similar irradiation modality in all studies supporting the “adaptive response”. Motivated by these curious discrepancies, we performed irradiation experiments on living mice, explanted mouse livers and liver homogenates observing that, in all conditions the activity of all these enzymes remained almost unchanged except for a slight increase found in explanted livers. Our results put a question about many previous scientific reports in this field.

## Introduction

Radiotherapy is used to treat localized solid tumors (skin, liver, brain, breast, etc.), and also leukemia and lymphoma. Over 50% of cancer patients are treated by radiotherapy at some stage of their illness^1^. Nowadays, radiation biology focuses primarily to understand the effects of the radiation on the cellular and micro-environmental systems^2,3^. The use of radiotherapy for the treatment of hepatic tumors is limited by the hepatic tolerance and the risk of radiation-induced liver damages. The development of advanced radiotherapy techniques (e.g., stereotactic body radiotherapy, which deliver high doses of radiotherapy in a single or small number of fractions), has enabled increasing use of radiotherapy to treat hepatocellular carcinoma^4–6^. However, a number of patients undergoing radiotherapy displays a range of side effects, which may lead to an interruption of treatment or limiting the dose of radiation. In fact, irradiation of normal tissues induces a cascade of events including oxidative stress eventually producing alteration of biological functions^7^. Following irradiation a chronic inflammatory healing response (from months to years) generating vascular and parenchymal cell dysfunction is observed^8^. Usually, the inflammatory response involves activation of kinases, transcription factors and production of inflammatory cytokines^8^. Furthermore, free radicals and reactive oxygen species are generated in the cell after irradiation inducing the formation of oxidized products. Irradiation consequences in vivo result in a rapid burst of reactive oxygen species, reactive nitrogen oxide species, and also oxidative stress and/or nitrosative stress^8^. The physiological manifestations of these radiation-induced alterations in redox sensitive processes (e.g., redox sensitive signaling pathways, transcription factor activation, and gene expression) have been suggested to contribute to inflammation, fibrosis and cytotoxicity.

At the matter of the facts, two lines of cell defense have been proposed: an “exogenous” protection that is represented by the protective role of natural and synthetic compounds against radiation and an “endogenous” one that is the “adaptive response”. Many compounds studied in radiotherapy to minimize the deleterious effects of ionizing irradiations derive essentially by natural products, such as flavonoids, phenylpropanoids, polyphenols, ascorbic acid, and gallic acid, and act as antioxidants, free radical scavengers, cytoprotective and radioprotective molecules^9^. The beneficial effects of these compounds reported in the recent literature stimulate the research to develop novel phytochemicals as radioprotectors for clinical use.

On the other hand, many endogenous defense mechanisms have evolved to minimize genotoxic damage, one of them is the “adaptive response”. This could be considered as a nonspecific phenomenon; the exposure to minimal stress (radiation) inducing a very low level of damage can trigger an “adaptive response” resulting in increased resistance to higher levels of the same or of other types of stress^10^.

In this context, it has been proposed that cells may protect themselves from oxidative products by hyper-expressing antioxidant enzymes like superoxide dismutase (SOD), catalase (CAT), glutathione-S-transferase (GST), glutathione reductase (GR), glutathione peroxidase (GPx), and other enzymes^11^. However, it is lacking a comparative and reasoned analysis of the many preceding studies about the effect of ionizing irradiations on the activity of the five of most studied antioxidant enzymes in mouse liver (GST, CAT, GR, SOD, and GPx).

In order to fill this gap, we deeply examined the present literature and then planed a number of irradiation experiments not only on living mice but even on explanted livers as well as on liver homogenates.

## Results

**Fig. 1:**
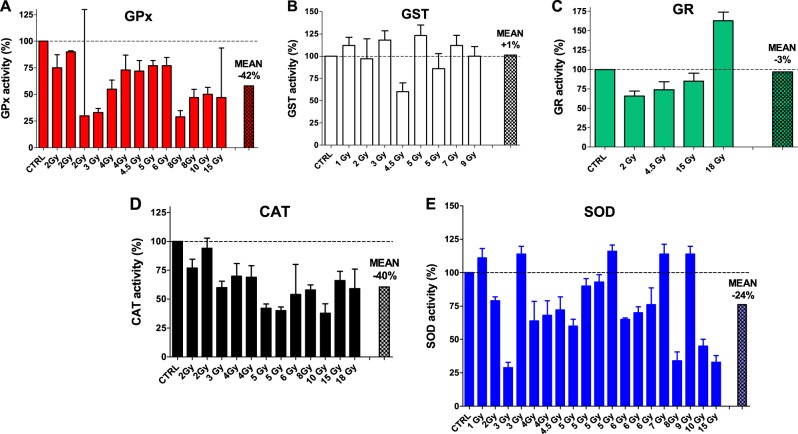
Effect of irradiation on the activity of antioxidant enzymes in liver as reported by several studies finalized to demonstrate the protective role of natural or synthetic drugs against irradiation. Percentage change ± SD of (**a**) glutathione peroxidase (GPx) 2 Gy^S1-S3^, 3 Gy^S4^, 4 Gy^S1,S5^, 4.5 Gy^S6^, 5 Gy^S7^, 6 Gy^S2^, 8 Gy^S1,S8^, 10 Gy^S2^, 15 Gy^S9^ (**b**) glutathione-S-transferase (GST) 1 Gy^S10^, 2Gy^S3^, 3 Gy^S10^, 4.5 Gy^S6^, 5 Gy^S10,S11^, 7 and 9 Gy^S10^ (**c**) glutathione reductase (GR) 2 Gy^S3^, 4.5 Gy^S6^, 15 Gy^S9^, 18 Gy^S12^ (**d**) catalase (CAT) 2 Gy^S2,S3^, 3 Gy^S4^, 4 Gy^S5,S13^, 5 Gy^S14,S15^, 6 Gy^S2^, 8 Gy^S8^, 10 Gy^S2^, 15 Gy^S9^, 18 Gy^S12^ and (**e**) superoxide dismutase (SOD) 1 Gy^S10^, 2 Gy^S2^, 3 Gy^S4,S10^, 4 Gy^S5,S13^, 4.5 Gy^S6^, 5 Gy^S7,S10,S14,S15^, 6 Gy^S2,S11,S16^, 7 Gy^S10^, 8 Gy^S8^, 9 Gy^S10^, 10 Gy^S2^, 15 Gy^S9^ respect to unirradiated controls (CTRL). The dashed lines represent the unirradiated controls (See Materials and Methods). The mean value is also shown for each panel. All References are whom reported in the Supplementary Information

In a first phase, we perused many previous articles describing mouse liver irradiation and finalized to discover radioprotective compounds able to minimize the induced damage on key antioxidant enzymes^S1-S16^. Data from these studies revealed that liver GPx, in irradiated mice (from 2 to 15 Gy) and without administered protective drugs, is inhibited i.e., under the level of unirradiated control mice, with an average activity of 58% (i.e., an average decreased activity of −42%) (Fig. 1a). The GST activities (after irradiation from 1 to 9 Gy) are approximately at the same level of the unirradiated controls (Fig. 1b) (except for the loss observed after 4.5 and 5 Gy), with an average value for all measurements of 101%. The effects of irradiations on GR were also studied; the activities after irradiation (from 2 to 15 Gy) are all below the control value (mean = 75%) but it increases after 18 Gy irradiation (160%) (Fig. 1c). Overall, the global average is close to unirradiated animals (97%). CAT behaves like GPx; after irradiations from 2 to 18 Gy all GPx activities are under the level of control mice with an average activity of 61% (an average decreased activity of −39%) (Fig. 1d). SOD, the most studied enzyme of this group, shows a lowered average activity of 76% after variable irradiations (from 1 to 15 Gy) (Fig. 1e).

In conclusion, in the absence of radioprotecting compounds, four of these five enzymes are inhibited at various extent up to 15 Gy irradiation while the only GST remained almost stable.

**Fig. 2:**
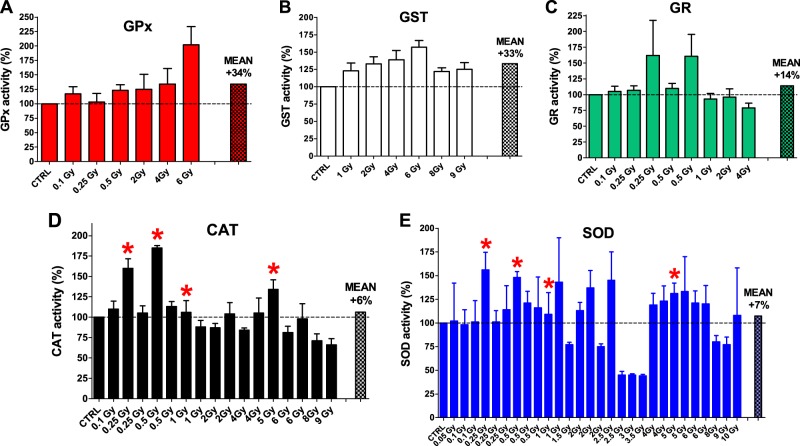
Effect of irradiation on activity of antioxidant enzymes in liver as reported by several studies supporting the “adaptive response” against irradiation. Percentage change ± SD of (**a**) glutathione peroxidase (GPx) 0.1, 0.25 and 0.5 Gy^S17^, 2, 4 and 6 Gy^S18^ (**b**) glutathione-S-transferase (GST) 1, 2, 4, 6, 8 and 9 Gy^S19^ (**c**) glutathione reductase (GR) 0.1 Gy^S17^, 0.25 and 0.5 Gy^S17,S20^, 1 and 2 Gy^S20^, 4 Gy^S21^ (**d**) catalase (CAT) 0.1 Gy^S17^, 0.25 and 0.5 Gy^S17,S22^, 1 Gy^S19,S22^, 2 and 4 Gy ^S18,S19^, 5 Gy^S22^, 6 Gy^S18,S19^, 8 and 9 Gy^S19^ and (**e**) superoxide dismutase (SOD) 0.05 Gy^S23^, 0.1 Gy ^S17,S23^, 0.25 and 0.5 Gy^S17,S22,S23^, 1 Gy^S22,S23^, 1.5 Gy^S24^, 2 Gy^S18,S19,S24^, 2.5 Gy^S23,S24^, 3 and 3.5 Gy^S24^, 4 Gy^S18,S19^, 5 Gy^S22,S23^, 6 Gy^S18,S19^, 8 and 9 Gy^S19^, 10 Gy^S23^ respect to unirradiated controls (CTRL). The dashed lines represent the unirradiated controls (See Materials and Methods). Red asterisks indicate activity values from irradiated explanted liver^S22^. The mean value is also shown for each panel. All References are whom reported in the Supplementary Information

The second group of studies is represented by articles proposing the “adaptive response” mechanism triggered by ionizing irradiations^S17-S24^. Data from unirradiated (control) and irradiated living mice (from 0.1 to 6 Gy) show that the GPx activities in liver are higher than the control with an average of 134% (Fig. 2a). The same tendency is found for GST activity (from 1 to 9 Gy) which increased by 33% (Fig. 2b). The GR activities (from 0.1 to 4 Gy) are almost similar to the control (except two cases at 0.25 and 0.5 Gy) with an overall average of 114% (Fig. 2c). The CAT activities (from 0.1 to 9 Gy) are scattered but the average is again similar to the control value (average is 106%) (Fig. 2d). The values of SOD, the most studied enzyme of this group, are scattered around control with an average of 107% after irradiations from 0.05 to 10 Gy (Fig. 2e). In conclusion, in the “adaptive response” class of articles, three enzymes display enhanced average activities after irradiation (GPx, GST and GR) and only a slight increase for CAT and SOD as reported in Fig. 2.

Finally in our third literature search, the mRNA levels are derived only from a few number of articles^S18,S25-S28^ with no variations respect to unirradiated control mice; only for GST isoform A3–3 and GR the values of transcripts are higher than controls (Fig. S1).

Taking into account the majority of the examined studies in literature, it is evident the curious discrepancy between the loss of activity of these enzymes found in the first paper group (studies showing the radio-protection by some drugs) and the increase of activity in the second paper group (studies describing the “adaptive response”) despite similar irradiation conditions.

**Fig. 3:**
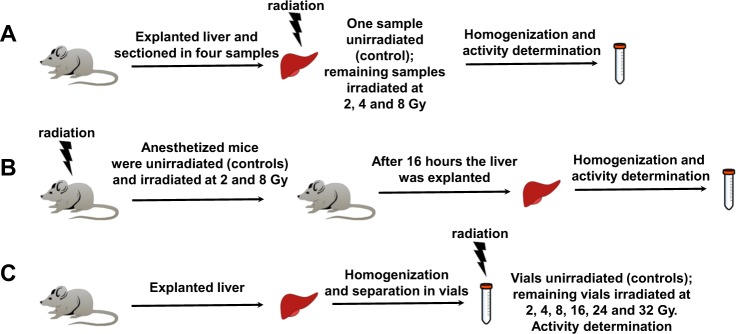
Schematization of irradiation experiments. **a** Explanted livers from mice were unirradiated (controls) and irradiated and finally the homogenates were analyzed for enzymatic activities. **b** Anesthetized mice were unirradiated (controls) and irradiated on the upper abdomen and then livers were explanted, homogenized and analyzed. **c** The explanted mouse livers were homogenized and the solutions unirradiated (controls) and irradiated. Finally, the enzymatic activities were measured. (Pictures are available free on-line at the website http://cliparts101.com/)

**Fig. 4:**
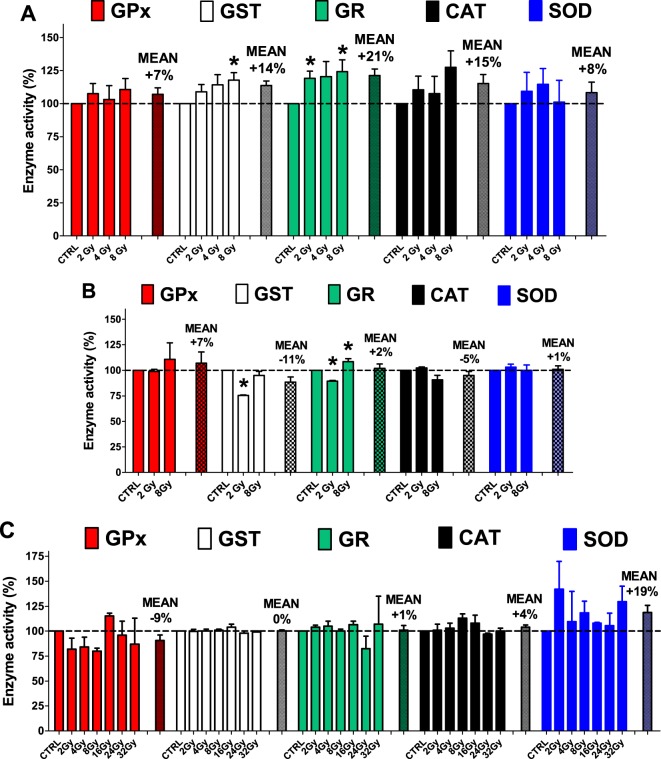
Antioxidant enzymes activities in mouse liver of after irradiation. Irradiation experiments (see Fig. 3 and Materials and Methods for details) on (**a**) explanted mouse livers (*N* = 8), (**b**) living mice (*N* = 4) and (**c**) mouse liver homogenates (*N* = 4). In all panels glutathione peroxidase (GPx) red bars, glutathione-S-transferase (GST) white bars, glutathione reductase (GR) green bars, catalase (CAT) black bars and superoxide dismutase (SOD) blue bars are reported. Each enzyme datasets are the activity levels obtained respect to unirradiated control (CTRL). Each bar represents an average ± standard error of the mean (SEM) of independent experiments. Means (±SEM) are also reported for each enzymes dataset. Results for the statistical analysis are reported in Table S1. Asterisks (*) indicate the observed differences between irradiated and CTRL samples (**a** *P* = 0.0152 GST 8 Gy *vs* control; *P* = 0.0103 GR 2 Gy *vs* control; *P* = 0.0291 GR 8 Gy *vs* control) (**b** *P* = 0.0125 GST 2 Gy *vs* control; *P* = 0.0341 GR 2 Gy *vs* control; *P* = 0.0443 GR 8 Gy *vs* control)

Thus, we decided to perform experiments at clinical radiation dose (from 2 to 8 Gy) on mouse liver considering that in tumor radiotherapy, the radiation dose is mainly based on the maximum dose tolerated by the normal tissue surrounding the target volume. Fig. 3 summarizes the three different type of experiments on explanted livers, anesthetized mice, and liver homogenates. The results of our experiments are in the three panels of Fig. 4. The enzyme activities found in irradiated explanted livers (from 2 to 8 Gy) show a statistically significant increase for GST (8 Gy) and GR (at 2 and 8 Gy) (from +19 to 24%) and a slight enhancement (not significative) for GPx, CAT and SOD at different Gy (Fig. 4a and Table S1). On the contrary, the anesthetized irradiated mice (Fig. 3b) show values of activities almost unchanged when compared to the unirradiated controls (Fig. 4b) except for modest but statistically significant decrease of GST (at 2 Gy) and GR (at 2 Gy) and a slight increase at 8 Gy for GR. In addition to clinical irradiation doses the liver homogenates were also irradiated at high-dose (up to 16, 24, and 32 Gy), but even in these conditions, distant from clinical recommendations, the enzymes solubilized in the homogenates showed no significant variations of activities levels (Fig. 4c).

## Discussion

While many papers adopted a single irradiation procedure i.e., on liver of a living mouse, or on explanted liver etc. we measured the effect of ionizing irradiation using three different modalities at the same time i.e., with liver explanted before irradiation, with liver irradiated in a living mouse and with liver explanted and homogenized before irradiation. Moreover, while most of the preceding studies in this field only reported the effect on the activity of one or two of the detoxifying enzymes, our data represent the entire panel of the five most important anti-oxidant enzymes.

In conclusion, all tested antioxidant enzymes appear resistant against irradiation treatments in living organism, explanted tissues, and homogenates. Our results point out that strong inactivation reported in scientific literature represent unintelligible results. Our data confirm a slight increase of cellular antioxidant defense only in irradiated explanted livers, and this phenomenon may be referred to an radiation-induced “adaptive response”^7^. However, the absence of this phenomenon in the liver of a living mouse suggests that peroxides, free radicals and other toxic compounds generated inside the cell by irradiations are probably eliminated and excreted by the hematic flux without triggering the hyper-expression of detoxifying enzymes. In the explanted liver this sweeping likely does not occur and the “adaptive response” becomes active. Our data, obtained on mice, cannot be referred to a living man, but they represent just a first indication that a typical clinical liver irradiation protocol to human patients could not alter the pool of active form of antioxidant enzymes as observed in the living mouse.

## Materials and Methods

### Chemicals

L-glutathione (GSH), oxidized glutathione (GSSG), 1-chloro-2,4-dinitrobenzene (CDNB), nicotinamide adenine dinucleotide phosphate in the reduced form (NADPH), 4-nitro blue tetrazolium chloride (NBT),  sodium azide (NaN_3_), hydrogen peroxide (H_2_O_2_), glutathione reductase (GR) from the baker’s yeast (*S. cerevisiae*), IGEPAL CA-630, ethylenediaminetetraacetic acid (EDTA), bovine serum albumin (BSA) and all other reagents were from SIGMA-Aldrich (St. Louis, Mo, USA). Bradford protein assay reagent (Bio-Rad). Protease inhibitor cocktail for use with mammalian cell and tissue extract from SIGMA-Aldrich (St. Louis, Mo, USA).

### Animals

Fourteen week old female CD1 mice (Envigo) were kept in the Interdepartmental Service Centre—Station for Animal Technology, University of Rome “Tor Vergata” (Italy) and housed at a constant temperature of 20 ± 2 °C, relative humidity of 50 ± 10%, on a 12/12 h light/dark cycle and ventilation 10–15 times/hours. Standard laboratory rodent pellet diet (4RF18; Mucedola srl, Italy) and water were provided to the animals ad libitum. All animal procedures were approved by Ethical Committee, conducted in accordance with national and international laws and policies (Italian Legislative Decree n. 116/92, now Italian Legislative Decree n. 26 4/3/14). Before all procedures mice were fully anesthetized with intraperitoneal injection of tiletamine/zolazepam (40 mg/kg) (Zoletil 100, Virbac, Italy) and xylazine (15 mg/kg) (Rompun, Bayer, Italy). Livers were explanted, divided in four parts, re-suspended in physiological solution and irradiated (Fig. 3a). For in vivo irradiation four animals in total were used. Mice were positioned on the left side and they have been irradiated for 3.5 min at 8 Gy and 2 Gy. After 16 h animals were sacrificed, the liver has been removed, divided in four parts and re-suspended in physiological solution (Fig. 3b). In the last experiment four mice were sacrificed and livers were explanted for homogenates production (Fig. 3c).

### Irradiation protocol

The irradiation of biological tissue was performed at room temperature, using a linear accelerator (Elekta Precise®), with 6 MV photons at a dose rate of 6 Gy/min. Single fraction of radiation doses of 2, 4, 8, 16, 24, and 32 Gy respectively were delivered to the biological tissue depending on the experiment. For irradiation, test specimens were immersed in an aqueous gel cube. A planning computed tomography (CT) was obtained in order to calculate the dose distribution within the test specimens (the enzymes in hydrosaline solution and liver homogenates). The treatment plan consisted of 2 opposing fields and was calculated on treatment planning system Pinnacle version 9.8 (Philips Medical System, Andover, MA). The radiation dose homogeneity was estimated at 3%. For mouse irradiation, each mouse underwent a planning CT to delineate the target volume (the whole liver) and a treatment plan was developed to deliver a dose (Fig. S2).

### Sample preparations

In the two experiments of irradiation: (a) irradiation of explanted mouse liver and (b) irradiation of the upper abdomen in anesthetized mice followed by liver explant, about 0.5 g of explanted liver unirradiated or irradiated with 2, 4 or 8 Gy was homogenized. The liver was put in 5 ml of 0.1 M phosphate buffer, pH 7.4 containing protease inhibitor cocktail and immediately after, 1 ml of homogenized liver was centrifuged at 13,300 rpm for 3 min. The supernatants were transferred in tubes on ice ready for enzymatic activity measurements. In the case of irradiation experiments on mouse liver homogenates, about 1 g of mouse liver was homogenized in 12 ml of physiologic solution with 0.9% sodium chloride containing protease inhibitor cocktail and 2 ml centrifuged at 13,300 rpm for 5 min. The supernatant was transferred in four vials on ice ready for irradiation experiments at 2, 4, 8, 16, 24 and 32 Gy.

### Glutathione peroxidase activity

Activity of GPx in liver was determined with a spectrophotometric assay at 340 nm (25 °C): 2 μl of homogenate was incubated in 1 ml of 0.1 M phosphate buffer, pH 7.4 (EDTA 0.1 mM) with 0.1 mM of NaN_3_, 1 mM of GSH, 1 µL of GR and 0.1 mM of NADPH. After linearity was reached, immediately 0.1 mM of H_2_O_2_ was added^12^. Each activity was normalized to the amount of total protein content determined by Bradford assay^13^.

### Glutathione-S-transferase activity

Activity of GST in liver was determined spectrophotometrically as described previously^14^. Briefly, 5 µl of homogenate was diluted in 1 ml of 0.1 M phosphate buffer, pH 6.5 and incubated with 1 mM GSH and 1 mM of CDNB. The enzymatic activity was followed at 340 nm (25 °C). Each spectrophotometric determinations were subtracted by the spontaneous reaction of the two co-substrates (GSH and CDNB). Activity determinations were normalized to the amount of total protein content determined for each sample by Bradford assay^13^.

### Glutathione reductase activity

Activity of GR in liver was determined spectrophotometrically at 340 nm (25 °C) diluting 10 μl of homogenate in 0.1 M phosphate buffer, pH 7.4 with 0.1 mM NADPH and, after the reaction reached linearity, suddenly 1 mM of GSSG was added^15^. Activity measurements were normalized to the amount of total protein content determined for each sample by Bradford assay^13^.

### Catalase activity

Activity of CAT in liver was determined with a spectrophotometric assay at 240 nm (25 °C): 10 μl of homogenate was diluted in 1 ml of 0.05 M phosphate buffer, pH 7.0 (EDTA 0.1 mM) with 1 mM of H_2_O_2_ according to the standard procedure described previously^16^. Each activity was normalized to the amount of total protein content determined by Bradford assay^13^.

### Preparation of cell lysates

Cell pellets were resuspended in lysis buffer containing 10 mM Tris-HCl, pH 7.4, 5 mM EDTA, 150 mM NaCl, 0.5% IGEPAL CA-630 and protease inhibitor cocktail (Sigma–Aldrich, St. Louis, MO, USA). After 30 min incubation on ice, cell lysates were centrifuged at 17,000 rpm for 15 min. Then supernatants were used to measure the SOD1 activity.

### Measurement of superoxide dismutase-1

SOD1 activity was evidenced on non-denaturing 7.5% polyacrylamide gels by loading 50 µg of total protein extracts. After electrophoresis, the gel was incubated in NBT solution (2.5 mM) for 30 min in the dark with gentle shaking, followed by 30 min incubation with a solution containing 30 mM tetramethylenediamine and 10 µg/ml riboflavin. SOD1 activity was detected as the achromatic band on the violet-colored gel, obtained after light exposure^17^. Density of immunoreactive bands was calculated using the software Quantity one (Bio-Rad). Catalase or β-tubulin was used as loading control. Proteins were assayed by the method of Lowry^18^.

### Data analysis

The literature search was performed taking into account the following conditions: mouse as animal model, liver as target organ, whole body or extirpated liver irradiated, enzymatic activity limited in particular for the antioxidant enzymes GPx, GST, GR, CAT and SOD. Animal models for tumor(s), radiation resistant, and treated with natural and synthetic drugs were excluded. Only in few analysis of mRNA^S25,S26^ and “adaptive response” enzyme activity^S21,S23,S24^ results from rat liver were included. Experimental data from different authors were obtained digitalizing histograms and graphs and then calculated the percentage values respect the control group in each study. The digitalization was performed using GetData Graph Digitizer software (v2.24). The graphic and results visualization were obtained by GraphPad Prism (La Jolla, CA, USA). Statistical analysis was performed between data pairs with a t-test; n.s. in Table S1 indicates that the observed difference between irradiated samples and controls are not statistically significant. *P* < 0.05 defines statistical significant differences (GraphPad InStat (La Jolla, CA, USA)).

## Supplementary information

Supplemental Material

Tiff Image
