# Supplementary material for: The impact of ionizing irradiation on liver detoxifying enzymes. A re-investigation
Source: Cell Death Discov. 2019 Feb 8;5:66. doi: 10.1038/s41420-019-0148-8 (PMC6368569; doi:10.1038/s41420-019-0148-8)
Supplement: Supplementary file 1 — Supplemental Material [file 41420_2019_148_MOESM1_ESM.pdf]

Supplementary Information

Supplementary Table

**Table S1. Statistical analysis of the observed differences between irradiated and non-irradiated samples from three different experimental protocols.**

Explanted mouse livers

| Gy | GPx | GST | GR | CAT | SOD |
|---|---|---|---|---|---|
| 2 | n.s. | n.s. | $P = 0.0103$[a] | n.s. | n.s. |
| 4 | n.s. | n.s. | n.s. | n.s. | n.s. |
| 8 | n.s. | $P = 0.0152$[a] | $P = 0.0291$[a] | n.s. | n.s. |

Living mice

| Gy | GPx | GST | GR | CAT | SOD |
|---|---|---|---|---|---|
| 2 | n.s. | $P = 0.0125$[a] | $P = 0.0341$[a] | n.s. | n.s. |
| 8 | n.s. | n.s. | $P = 0.0443$[a] | n.s. | n.s. |

Mouse liver homogenates

| Gy | GPx | GST | GR | CAT | SOD |
|---|---|---|---|---|---|
| 2 | n.s. | n.s. | n.s. | n.s. | n.s. |
| 4 | n.s. | n.s. | n.s. | n.s. | n.s. |
| 8 | n.s. | n.s. | n.s. | n.s. | n.s. |
| 16 | n.s. | n.s. | n.s. | n.s. | n.s. |
| 24 | n.s. | n.s. | n.s. | n.s. | n.s. |
| 32 | n.s. | n.s. | n.s. | n.s. | n.s. |

[a] $P < 0.05$ is considered statistically significant.
n.s. is not-significant

Supplementary Figure 1

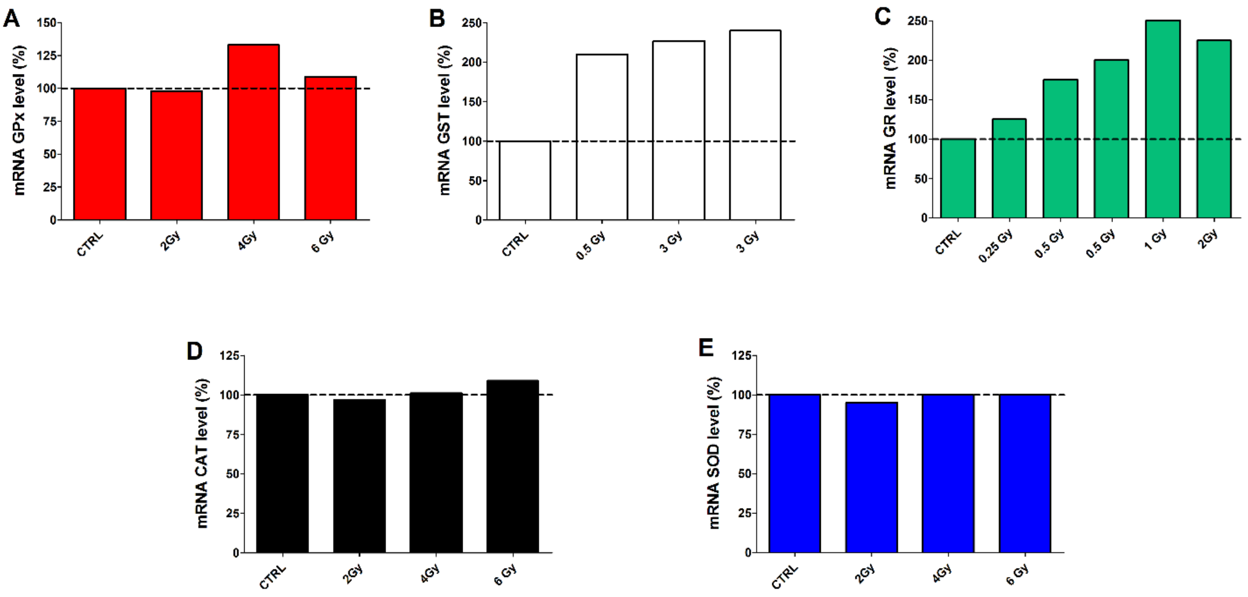


**Figure S1. Effect of irradiation on transcripts of different antioxidant enzymes.** Percentage change of mRNA expression levels of (**a**) glutathione peroxidase (GPx) 30 min after irradiation at 2, 4 and 6 Gy[S18] (**b**) glutathione-S-transferase (GST isoform A3-3) 12 hours after irradiation at 0.5 Gy[S25], 3 Gy[S25,S26] (**c**) glutathione reductase (GR) 3 hours after irradiation at 0.25 Gy[S27], 0.5 Gy[S27,S28], 1 and 2 Gy[S27] (**d**) catalase (CAT) 30 min after irradiation at 2, 4 and 6 Gy[S18] and (**e**) superoxide dismutase (SOD) 30 min after irradiation at 2, 4 and 6 Gy[S18] respect to unirradiated controls (CTRL). The dashed lines represent the unirradiated controls (See Materials and Methods). All References are whom reported in the present Supplementary Information section (see below).

Supplementary Figure 2

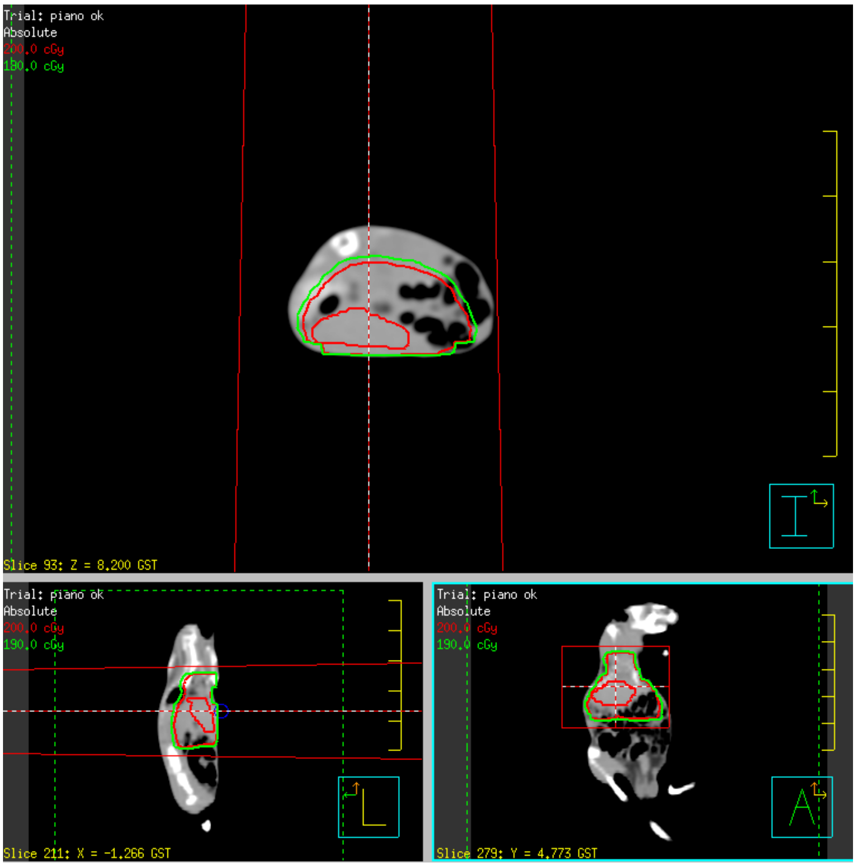


**Figure S2. Computed tomography (CT) of an irradiated mouse.** On the top, the axial image of the mouse CT showing the liver (contoured in red) in the abdomen. The wider red line and the green line represent the 95% and the 90% isodose of the prescribed dose. In the bottom left, the sagittal reconstruction. In the bottom right, the coronal reconstruction.

Supplementary References


S1. Nair, G. G. & Nair, C. K. Radioprotective effects of gallic acid in mice. *BioMed Res. Int.* **2013**, 953079 (2013).

S2. Sandeep, D. & Nair, C. K. Protection from lethal and sub-lethal whole body exposures of mice to γ-radiation by Acorus calamus L.: studies on tissue antioxidant status and cellular DNA damage. *Exp. Toxicol. Pathol.* **64**, 57-64 (2012).

S3. Koul, A. & Abraham, S. K. Intake of saffron reduces γ-radiation-induced genotoxicity and oxidative stress in mice. *Toxicol. Mech. Methods* **27**, 428-434 (2017).

S4. Tawfik, S. S., Abouelella, A. M. & Shahein, Y. E. Curcumin protection activities against γ-rays-induced molecular and biochemical lesions. *BMC Res. Notes* **6**, 375 (2013).

S5. Smina, T. P., Joseph, J. & Janardhanan, K. K. Ganoderma lucidum total triterpenes prevent γ-radiation induced oxidative stress in Swiss albino mice in vivo. *Redox Rep.* **21**, 254-261 (2016).

S6. Devi, P. U. & Ganasoundari, A. Modulation of glutathione and antioxidant enzymes by Ocimum sanctum and its role in protection against radiation injury. *Indian J. Exp. Biol.* **37**, 262-268 (1999).

S7. Zhao, H. et al. Protective effect of anthocyanin from Lonicera Caerulea var. Edulis on radiation-induced damage in mice. *Int. J. Mol. Sci.* **13**, 11773-11782 (2012).

S8. Shanthakumar, J., Karthikeyan, A., Bandugula, V. R. & Rajendra Prasad, N. Ferulic acid, a dietary phenolic acid, modulates radiation effects in Swiss albino mice. *Eur. J. Pharmacol.* **691**, 268-274 (2012).

S9. Kim, H. G., Jang, S. S., Lee, J. S., Kim, H. S. & Son, C. G. Panax ginseng Meyer prevents radiation-induced liver injury via modulation of oxidative stress and apoptosis. *J. Ginseng Res.* **41**, 159-168 (2017).

S10. Chandra, D. & Kale, R. K. Influence of gamma-rays on the mouse liver cytochrome P450 system and its modulation by phenothiazine drugs. *Int. J. Radiat. Biol.* **75**, 335-349 (1999).

S11. Pan, J. et al. In Vivo Radioprotective Activity of Cell-Permeable Bifunctional Antioxidant Enzyme GST-TAT-SOD against Whole-Body Ionizing Irradiation in Mice. *Oxid. Med. Cell Longev.* **2017**, 2689051 (2017).

S12. Neal, R., Matthews, R. H., Lutz, P. & Ercal, N. Antioxidant role of N-acetyl cysteine isomers following high dose irradiation. *Free Radic. Biol. Med.* **34**, 689-695 (2003).

S13. Ran, Y. et al. Dragon's blood and its extracts attenuate radiation-induced oxidative stress in mice. *J. Radiat. Res.* **55**, 699-706 (2014).

S14. Sinha, M. et al. Epicatechin ameliorates ionising radiation-induced oxidative stress in mouse liver. *Free Radic. Res.* **46**, 842-849 (2012).

S15. Sinha, M., Das, D. K., Bhattacharjee, S., Majumdar, S. & Dey, S. Leaf extract of Moringa oleifera prevents ionizing radiation-induced oxidative stress in mice. *J. Med. Food* **14**, 1167-1172 (2011).

S16. Ping, X., Junqing, J., Junfeng, J. & Enjin, J. Radioprotective effects of troxerutin against gamma irradiation in mice liver. *Int. J. Radiat. Biol.* **88**, 607-612 (2012).

S17. Avti, P. K. et al. Low dose gamma-irradiation differentially modulates antioxidant defense in liver and lungs of Balb/c mice. *Int. J. Radiat. Biol.* **81**, 901-910 (2005).

S18. Hardmeier, R., Hoeger, H., Fang-Kircher, S., Khoschsorur, A. & Lubec G. Transcription and activity of antioxidant enzymes after ionizing irradiation in radiation-resistant and radiation-sensitive mice. *Proc. Natl. Acad. Sci. USA* **94**, 7572-7576 (1997).

S19. Agrawal, A., Chandra, D. & Kale, R. K. Radiation induced oxidative stress: II studies in liver as a distant organ of tumor bearing mice. *Mol. Cell Biochem.* **224**, 9-17 (2001).

S20. Kojima, S. et al. Does small-dose gamma-ray radiation induce endogenous antioxidant potential in vivo? *Biol. Pharm. Bull.* **20**, 601-604 (1997).

S21. Mortazavi, S. M. J. et al. Adaptive response Induced by Pre-Exposure to 915 MHz Radiofrequency: A Possible Role for Antioxidant Enzyme Activity. *J. Biomed Phys. Eng.* **7**, 137-142 (2017).

S22. Kataoka, T. et al. Basic study on active changes in biological function of mouse liver graft in cold storage after low-dose x-irradiation. *J. Clin. Biochem. Nutr.* **45**, 219-226 (2009).

S23. Yamaoka, K., Edamatsu, R. & Mori, A. Increased SOD activities and decreased lipid peroxide levels induced by low dose X irradiation in rat organs. *Free Radic. Biol. Med.* **11**, 299-306 (1991).

S24. Zakaria, K. M. Effect of Gamma Ray on Reactive Oxygen Species at Experimental Animals. *OMICS J. Radiol.* **6**, 283 (2017).

S25. Kim, S. G. et al. Enhancement of radiation-inducible hepatic glutathione-S-transferases Ya, Yb1, Yb2, Yc1, and Yc2 gene expression by oltipraz: possible role in radioprotection. *Mol. Pharmacol.* **51**, 225-233 (1997).

S26. Nam, S. Y., Cho, C. K. & Kim, S. G. Correlation of increased mortality with the suppression of radiation-inducible microsomal epoxide hydrolase and glutathione S-transferase gene expression by dexamethasone: effects on vitamin C and E-induced radioprotection. *Biochem. Pharmacol.* **56**, 1295-1304 (1998).

S27. Kojima, S. et al. Induction of mRNAs for glutathione synthesis-related proteins in mouse liver by low doses of gamma-rays. *Biochim. Biophys. Acta* **1381**, 312-318 (1998).

S28. Kojima, S., Matuski, O., Nomura, T., Takahashi, M. & Yamaoka, K. Effect of small doses of γ-ray on the glutathione synthesis in mouse (IAEA-TECDOC--976). International Atomic Energy Agency (IAEA) (1997).
