# Supplementary figures and images for: The impact of ionizing irradiation on liver detoxifying enzymes. A re-investigation
Source: Cell Death Discov. 2019 Feb 8;5:66. doi: 10.1038/s41420-019-0148-8 (PMC6368569; doi:10.1038/s41420-019-0148-8)

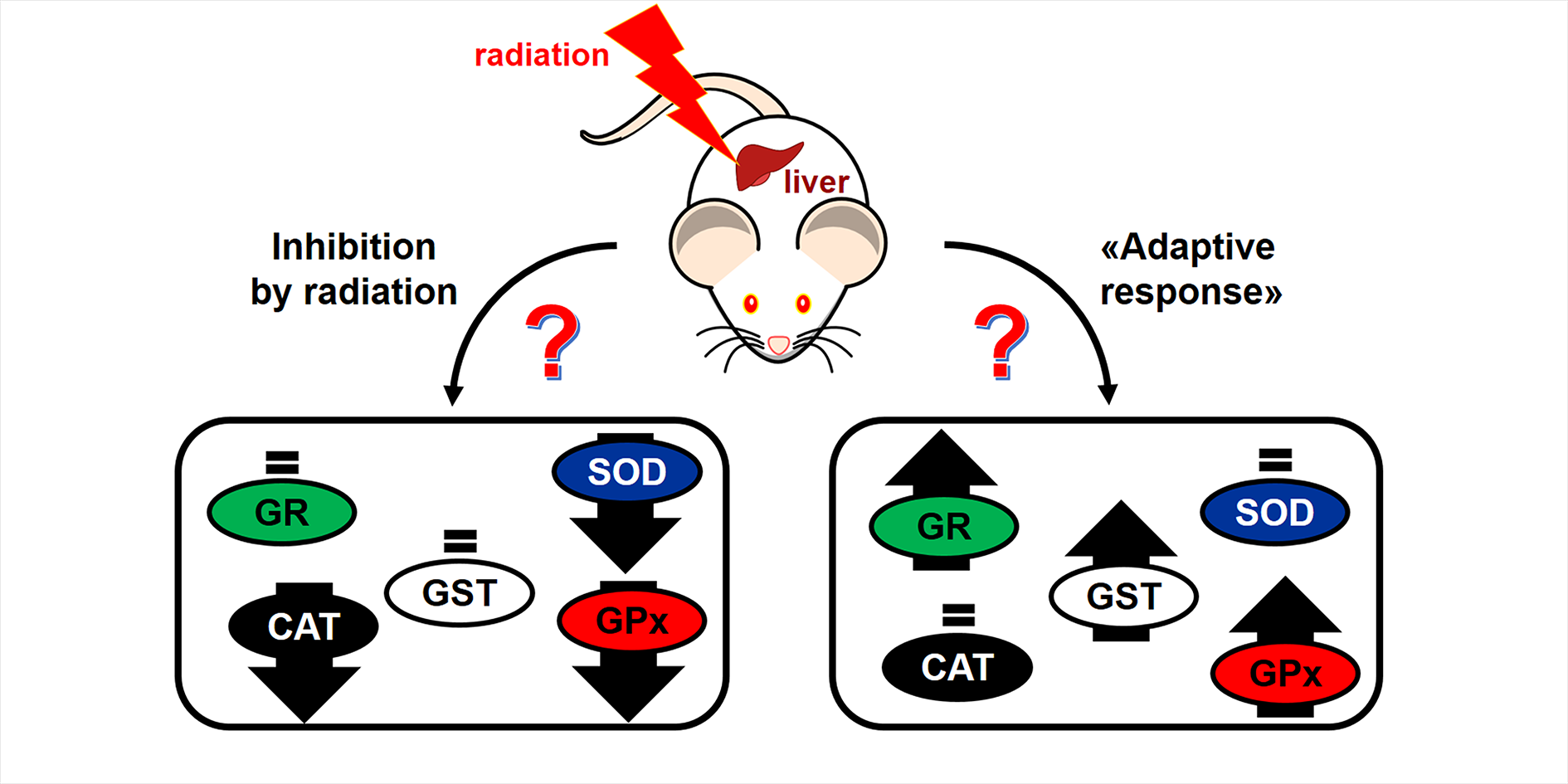

Supplement: Supplementary file 2 — Tiff Image [file 41420_2019_148_MOESM2_ESM.tif]
